# Correlation of High Seawater Temperature with *Vibrio* and *Shewanella* Infections, Denmark, 2010–2018

**DOI:** 10.3201/eid2903.221568

**Published:** 2023-03

**Authors:** Yaovi Mahuton Gildas Hounmanou, Jørgen Engberg, Karsten Dalsgaard Bjerre, Hanne Marie Holt, Bente Olesen, Marianne Voldstedlund, Anders Dalsgaard, Steen Ethelberg

**Affiliations:** University of Copenhagen, Copenhagen, Denmark (Y.M.G. Hounmanou, A. Dalsgaard, S. Ethelberg);; Zealand University Hospital, Køge, Denmark (J. Engberg);; Statens Serum Institut, Copenhagen (K.D. Bjerre, M. Voldstedlund, S. Ethelberg);; Odense University Hospital, Odense, Denmark (H.M. Holt);; Herlev and Gentofte University Hospital, Herlev, Denmark (B. Olesen).

**Keywords:** *Vibrio*, *Shewanella*, seawater temperature, Denmark, infections, climate change, bacteria

## Abstract

During 2010–2018 in Denmark, 638 patients had *Vibrio* infections diagnosed and 521 patients had *Shewanella* infections diagnosed. Most cases occurred in years with high seawater temperatures. The substantial increase in those infections, with some causing septicemia, calls for clinical awareness and mandatory notification policies.

*Vibrio* and *Shewanella* spp. bacteria cause a variety of human infections, including wound infections, ear infections, septicemia, and gastroenteritis (*1*). Domestically acquired *Vibrio* and *Shewanella* infections occur only sporadically in countries in northern Europe because the coastal seawater temperature tends to be too cold to support growth and high bacterial pathogen concentration levels (*2*,*3*). However, the warming of low-salinity coastal waters of the Baltic Sea has promoted the growth of *Vibrio* and *Shewanella* spp. and consequently increased the risk of disease for humans exposed to such seawater (*4*). In the unusually warm summer of 1994 in Denmark, several *V. vulnificus* and *S. algae* infections were seen among patients who reported bathing in seawater (*5*,*6*). Furthermore, during 2014–2018, more than 1,055 cases of vibriosis were reported in northern Europe countries, including Denmark (*7*).

Considering the annual increase in infections during recent summer seasons in Denmark and the recurring heatwaves across Europe, this emerging public health threat requires more investigation to provide decision-makers with evidence for action. The aim of our nationwide study was to describe the distribution of *Vibrio* and *Shewanella* infections in Denmark during 2010–2018 and investigate a possible correlation between infections and sea surface temperature.

## The Study

We studied cases of *Vibrio* and *Shewanella* infections during 2010–2018 in the summer months in Denmark (June to August); in the decade spanning 2010–2020, 2018 was the warmest registered summer in the country. We obtained information about the cases from the Danish Microbiology Database, a national database containing all clinical microbiology reports from Denmark (*8*). We extracted identification results, confirmed by matrix-assisted laser desorption/ionization time-of-flight mass spectrometry, on *Vibrio* and *Shewanella* spp. cultured from blood, wound swabs, deep soft tissue, ear, trachea, urine, and feces as well as information about date of sampling. We also extracted the person identification number of each patient studied from Denmark’s Central Person Registry (CPR) (*9*). Clinical patient information was not available, but sample types were used as a proxy for type of infection. We registered cases by month per patient (Appendix 1). We counted the number of cases by calendar year and stratified them by the genus of isolated bacterial pathogen. Using the CPR number, we eliminated duplicate positive results. By linking to data from the CPR register, we retrieved information on address of residence for each case at the time of sampling. We performed geomapping and geocoding in QGIS 1.8.0 Lisboa (https://www.qgis.org) for the spatial analysis of *Shewanella* and *Vibrio* cases and plotting of number of infections per municipality, which we further interpreted based on seawater salinity in the mapped areas. We obtained sea surface temperatures of the coastal waters of Denmark during summer from the Danish Meteorological Institute (Appendix 1; Appendix 2). We performed the Pearson correlation test in R version 4.2.1 (The R Foundation for Statistical Computing, https://www.r-project.org) to determine correlation between annual summer seawater temperatures and number of *Vibrio* and *Shewanella* cases.

**Figure 1:**
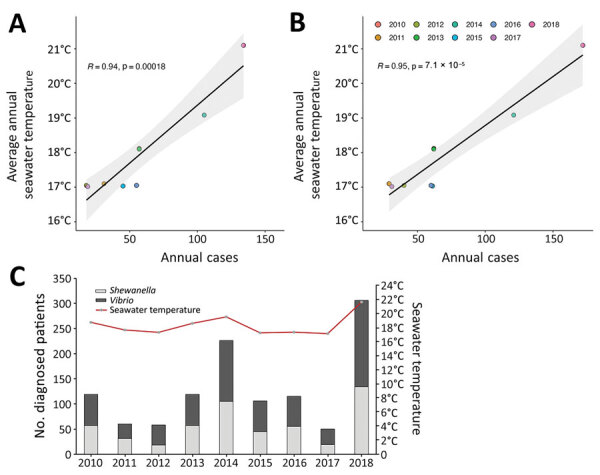
Relationship between *Shewanella *and *Vibrio* spp. infections and seawater temperature in Denmark, by year, 2010–2018. A, B) Pearson correlation between annual numbers of diagnosed *Shewanella *(A) and *Vibrio* (B) infections. C) Comparison of *Shewanella* and *Vibrio* cases and average summer seawater temperature by year.

We found a positive correlation between average summer seawater temperatures (15°C–22°C) and the number of cases of *Vibrio* (29–172) and *Shewanella* (18–134) infections diagnosed in Denmark during 2010–2018 (p<0.0001; Figure 1, panels A, B). Results showed a higher number of infections during warmer summers compared with colder summers. Ear infections (n = 595) and wound infections (n = 424) were the most frequent clinical manifestations (Table); *V. alginolyticus* and *S. algae* were predominant in ear infections. *V. parahaemolyticus* was the most frequently isolated from wounds (n = 103, 24%), *V. vulnificus* (n = 14, 36%) and *S. putrefaciens* (n = 10, 26%) were predominant in septicemia cases, and *S. putrefaciens* was the species most associated with deep soft tissue infections (Table). Clinical manifestations varied by bacterial species. More than one third of *V. vulnificus* infections manifested as septicemia, supporting evidence of the high virulence of this species (*10*–*12*). 

**Table:** Distribution of species per type of *Vibrio* and *Shewanella* infections, Denmark 2010–2018*

Bacterial species	Type of manifestation or site of infection, no. (%)								
	Ear	Wound and shallow soft tissue	Septicemia	Feces†	Deep soft tissue	Respiratory tract	Urine	Other
*Shewanella algae*	99 (16.6)	82 (19.3)	4 (10.3)	NA	3 (12.5)	6 (30)	1 (7.7)	2 (9.5)
*S. putrefaciens*	86 (14.5)	80 (18.9)	10 (25.6)	NA	12 (50)	7 (35)	8 (61.5)	10 (47.6)
*Shewanella* spp.‡	58 (9.7)	41 (9.7)	1 (2.6)	NA	1 (4.2)	6 (30)	2 (15.4)	2 (9.5)
Total *Shewanella*	243	203	15	NA	16	19	11	14
*Vibrio alginolyticus*	248 (41.7)	72 (17)	2 (5.1)	2 (8.7)	4 (16.7)	1 (5)	0	4 (19)
*V. cholerae*	12 (2)	1 (0.2)	0	4 (17.4)	0	0	0	1 (4.8)
*V. fluvialis*	9 (1.5)	3 (0.7)	1 (2.6)	4 (17.4)	0	0	0	0
*V. parahaemolyticus*	36 (6.1)	103 (24.3)	5 (12.8)	9 (39.1)	2 (8.3)	0	0	1 (4.8)
*V. vulnificus*	2 (0.3)	11 (2.6)	14 (35.9)	1 (4.3)	2 (8.3)	0	2 (15.4)	0
*Vibrio* spp.‡	45 (7.6)	31 (7.3)	2 (5.1)	3 (13)	0	0	0	1 (4.8)
Total *Vibrio*	352	221	24	23	8	1	2	7
Total	595	424	39	23	24	20	13	21

*NA, not applicable. †*S. algae, S. putrefaciens, S. spp.* found in feces samples (n = 24) were excluded from all analyses. ‡Not identified to species level.

*Vibrio *and *Shewanella* infections increased during every summer in the study period. The summers of 2014 and 2018 were characterized by particularly high sea surface temperatures and showed an association to higher incidence in infections (Figure 1, panels A, B). In all studied years, the frequency of *Vibrio* and *Shewanella* spp. infections increased beginning in week 23, reaching a peak in the warmest months (July and August), followed by a tail of decreasing number in subsequent months (Appendix 1 Figure, panel A). A recent study on 2018 data alone reported that most human vibriosis cases reported in the Nordic region were likely linked to exposure to the warm seawater that year (*7*). We found that infections were more prevalent in men and boys 10–19 years of age and in elderly persons, 60–80 years of age (Appendix 1 Figure, panel B). We suspect that those results are likely because active adolescents may have scratches or wounds while performing recreational water activities (e.g., swimming, rowing, windsurfing, or fishing) and because of the vulnerability of elderly persons in general.

**Figure 2:**
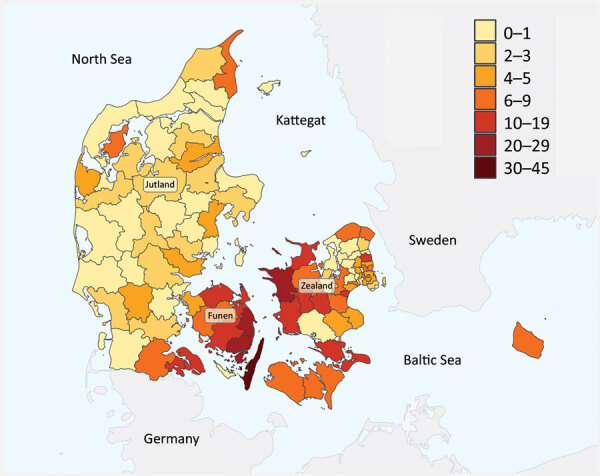
Number of cases of *Shewanella* and* Vibrio* spp. infection (n = 98), by municipality, Denmark, 2018.

We found a marked geographic distribution in results obtained from 2018, when most cases were in persons who lived near coastal areas with brackish waters characterized by low saline levels (<30 parts per thousand; Figure 2) (*13*). In contrast, along the west coast of Jutland, where the salinity of the North Sea is high and the water colder, the frequency of infections was lower. This difference suggests that increased temperature of low-salinity water favors the growth of *Vibrio* and *Shewanella* bacteria. It is important to consider that the association between place of residence and number of cases is challenged because geographic distances are short in Denmark and multiple exposures at different geographic sites during a summer season are to be expected. The lack of information on prior seawater exposure and information on international travel for each case is a limitation for the correlation between number of *Vibrio* and *Shewanella* infections and seawater exposure. Nevertheless, the observed geographic distribution of cases and the presented correlation between the number of cases in cold and warm summers strongly supports a relationship between higher-temperature/low-saline seawater exposure and risk of infection.

## Conclusions

In this nationwide study, we show a correlation between number of *Vibrio* and *Shewanella* human infections and coastal summer water temperature in Denmark during 2010–2018. In addition, we were able to map residency of most cases to geographic areas with coastlines of low salinity. A combination of climate change effects (i.e., increasing coastal sea surface temperature at higher latitudes during summer) and a more elderly population indicates the need for increased awareness of the risk of these emerging infections and their public health impact. Rising temperatures will lead to an increase in burden of disease for these marine infections in an expanding area of the northern hemisphere (*14*). We propose that persons in Denmark who are exposed to seawater in summer should consider covering open wounds with a waterproof bandage, particularly the elderly and immunocompromised. We also recommend that persons thoroughly wash new cuts exposed to seawater and inform healthcare professionals of recent seawater exposure when seeking medical attention. Persons with defected eardrums should use earplugs. Our study lends support to categorizing all *Vibrio* and *Shewanella* infections in humans as mandatory notifiable diseases in Denmark and other countries in Europe that have seawater borders to monitor the incidence of these infections.

Appendix 1 Description of materials and methods for correlation of high seawater temperature with* Vibrio* and *Shewanella* infections, Denmark, 2010–2018.

Appendix 2 Bacterial isolation procedure and average summer sea temperature for inner water sites for correlation of high seawater temperature with* Vibrio* and *Shewanella* infections, Denmark, 2010–2018.

## References

[1] Tantillo GM, Fontanarosa M, Di Pinto A, Musti M. Updated perspectives on emerging vibrios associated with human infections. Lett Appl Microbiol. 2004;39:117–26. 10.1111/j.1472-765X.2004.01568.x 15242449

[2] Baker-Austin C, Trinanes JA, Taylor NGH, Hartnell R, Siitonen A, Martinez-Urtaza J. Emerging *Vibrio* risk at high latitudes in response to ocean warming. Nat Clim Chang. 2013;3:73–7. 10.1038/nclimate1628

[3] Gram L, Bundvad A, Melchiorsen J, Johansen C, Fonnesbech Vogel B. Occurrence of *Shewanella algae* in Danish coastal water and effects of water temperature and culture conditions on its survival. Appl Environ Microbiol. 1999;65:3896–900. 10.1128/AEM.65.9.3896-3900.1999 10473392 PMC99717

[4] Baker-Austin C, Trinanes JA, Salmenlinna S, Löfdahl M, Siitonen A, Taylor NGH, et al. Heat wave-associated vibriosis, Sweden and Finland, 2014. Emerg Infect Dis. 2016;22:1216–20. 10.3201/eid2207.151996 27314874 PMC4918148

[5] Dalsgaard A, Frimodt-Møller N, Bruun B, Høi L, Larsen JL. Clinical manifestations and molecular epidemiology of *Vibrio vulnificus* infections in Denmark. Eur J Clin Microbiol Infect Dis. 1996;15:227–32. 10.1007/BF01591359 8740858

[6] Holt HM, Søgaard P, Gahrn-Hansen B. Ear infections with *Shewanella* alga: a bacteriologic, clinical and epidemiologic study of 67 cases. Clin Microbiol Infect. 1997;3:329–34. 10.1111/j.1469-0691.1997.tb00622.x 11864129

[7] Amato E, Riess M, Thomas-Lopez D, Linkevicius M, Pitkänen T, Wołkowicz T, et al. Epidemiological and microbiological investigation of a large increase in vibriosis, northern Europe, 2018. Euro Surveill. 2022;27:2101088. 10.2807/1560-7917.ES.2022.27.28.2101088 35837965 PMC9284918

[8] Voldstedlund M, Haarh M, Mølbak K; MiBa Board of Representatives. The Danish Microbiology Database (MiBa) 2010 to 2013. Euro Surveill. 2014;19:20667. 10.2807/1560-7917.ES2014.19.1.20667 24434175

[9] Schmidt M, Pedersen L, Sørensen HT. The Danish civil registration system as a tool in epidemiology. Eur J Epidemiol. 2014;29:541–9. 10.1007/s10654-014-9930-3 24965263

[10] Frank C, Littman M, Alpers K, Hallauer J. *Vibrio vulnificus* wound infections after contact with the Baltic Sea, Germany. Euro Surveill. 2006;11:E060817.1. 16966781 10.2807/esw.11.33.03024-en

[11] Li G, Wang MY. The role of *Vibrio vulnificus* virulence factors and regulators in its infection-induced sepsis. Folia Microbiol (Praha). 2020;65:265–74. 10.1007/s12223-019-00763-7 31840198

[12] Horseman MA, Surani S. A comprehensive review of *Vibrio vulnificus*: an important cause of severe sepsis and skin and soft-tissue infection. Int J Infect Dis. 2011;15:e157–66. 10.1016/j.ijid.2010.11.003 21177133

[13] Kniebusch M, Meier HEM, Radtke H. Changing salinity gradients in the Baltic Sea as a consequence of altered freshwater budgets. Geophys Res Lett. 2019;46:9739–47. 10.1029/2019GL083902

[14] Trinanes J, Martinez-Urtaza J. Future scenarios of risk of *Vibrio* infections in a warming planet: a global mapping study. Lancet Planet Health. 2021;5:e426–35. 10.1016/S2542-5196(21)00169-8 34245713

