# Supplementary material for: Correlation of High Seawater Temperature with Vibrio and Shewanella Infections, Denmark, 2010–2018
Source: Emerg Infect Dis. 2023 Mar;29(3):605–8. doi: 10.3201/eid2903.221568 (PMC9973676; doi:10.3201/eid2903.221568)
Supplement: Appendix 1 — Description of materials and methods for correlation of high seawater temperature with Vibrio and Shewanella infections, Denmark, 2010–2018. [file 22-1568-Techapp-s1.pdf]

Article DOI: http://doi.org/10.3201/eid2903.221568

EID cannot ensure accessibility for supplementary materials supplied by authors. Readers who have difficulty accessing supplementary content should contact the authors for assistance.

Correlation of High Seawater Temperature with *Vibrio* and *Shewanella* Infections, Denmark, 2010–2018

Appendix 1

Materials and Methods

Description of the study area and framework

Denmark is a Nordic European country consisting of a peninsula, Jutland, and an archipelago of 78 inhabited islands. The sea surrounding the major islands e.g., in the Baltic Sea, have saline levels of between 10–20 parts per thousand. Denmark has a population of 5.8 million and is divided into five administrative Regions, which are further subdivided into 98 municipalities. The administrative Regions are administrating the health care system, which is tax-funded. There are currently ten clinical microbiological laboratories throughout the country, responsible for all clinical microbiology diagnostic services. Clinical microbiology laboratory results have since 2010 been recorded systematically in the national Danish Microbiology Database (MiBa) (*1*). A unique person identification number (Central Person Registry, CPR) enables linkage between health and administrative register data and identifies all Danish residents (*2*).

Data extraction

The study period was from 2010 to 2018. From MiBa, we searched for and extracted information on all bacterial cultures done for *Vibrio* spp. and *Shewanella* spp. These bacterial culture data were registered in MiBa following standardized microbiological procedures across all clinical laboratories in the country (Appendix 2, Table 1). The extracted data included date of sampling; anatomic sampling site of the patient, CPR number of person tested and test results. The quality of data was validated by comparison with local clinical microbiology laboratory

databases at the Departments of Clinical Microbiology at Herlev and Gentofte Hospital, Odense University Hospital and Zealand University Hospital. This comparison confirmed that patients registered in the laboratory systems had also been correctly identified in MiBa (data not shown). Information on municipality of residence was obtained from the Central Person Registry (*2*). Demographic data, i.e., size of the Danish population by sex, age, municipality and year, were obtained from Statistics Denmark (*3*). As cases were extracted from the MiBa database, information on recent seawater exposure or foreign travel were not available.

Oceanographic data mainly seawater temperature characterizing the summer periods in Denmark (July to August) was obtained from the Danish Meteorological Institute (https://confluence.govcloud.dk/display/FDAPI/Oceanographic+Observation). All measurements performed within the study period were from all inner water sites, i.e., from the southwestern part of the Baltic Sea, the Belt Sea, the sound and Kattegat and were obtained at depths of 0.1 and 0.2 m. The average annual summer temperature was calculated, disregarding the time of the day of the measurement and the varying number of measurements from each seawater site (Appendix 2, Table 2).

Data analysis

The CPR number, allowing for elimination of duplicate positive test results, identified individual cases. *Shewanella* (*S. algae, S. putrefaciens, S.* species) found in feces samples (n = 24) was excluded from all analyses. We counted cases by calendar year and pathogen genus, i.e., if the same individual was found positive several times per year with either *Vibrio* or *Shewanella* this was recorded as one case only (Appendix 1 Table). If a person had more than one sample taken – then infection was counted by the first date. Pathogen genus is equivalent to the entries listed (Table). That is, a patient may e.g., have both a *Vibrio* spp. case and a *V. fluvialis* case within the same calendar year. In the cases of polymicrobial infections with additional non-*Vibrio/Shewanella* species, only the *Vibrio* and *Shewanella* were counted are presented in this study.

The Pearson correlation test was performed in R version 4.2.1 to determine the correlation between variations of annual summer seawater temperature on the annual number of cases of *Vibrio* and *Shewanella* infections.

Geo mapping and geocoding QGIS 1.8.0_Lisboa (www.qgis.org) was used for the spatial analysis of *Shewanella* and *Vibrio* cases and plotting of number of infections per municipality. A geographical database with

municipality borders in vector format (SHP file) was obtained from the Danish Geodata Agency (GST). Cases were geocoded using the Central Population Registry (CPR registry) and the geocoding of addresses from GST. The address data used for the study originated from the Danish Geodata Agency and were built on Official Standard Addresses and Coordinates (OSAK). Addresses were joined to cases based on the date of the date of sampling.

References

1. Voldstedlund M, Haarh M, Mølbak K; MiBa Board of Representatives. The Danish Microbiology Database (MiBa) 2010 to 2013. Euro Surveill. 2014;19:20667. PubMed https://doi.org/10.2807/1560-7917.ES2014.19.1.20667

2. Pedersen CB. The Danish Civil Registration System. Scand J Public Health. 2011;39(Suppl):22–5. PubMed https://doi.org/10.1177/1403494810387965

3. Statistics Denmark. Statistics Denmark [cited 2022 Jul 26]. https://www.dst.dk/en

Appendix 1 Table. Population flowchart

Source	Number of samples	Exclusion
epiMIBA extract (*Shewanella*, *Vibrio*)	1456	
Sample date 2010–2018, outside of interval		11
Organism not correctly identified		1
CPR No not valid		24
Shewanella in fecal samples		24
Sample type not relevant		10
Population I	1386	
Address not available in CPR		5
Population II	1381	
Number of cases (patient year organism)	1159	

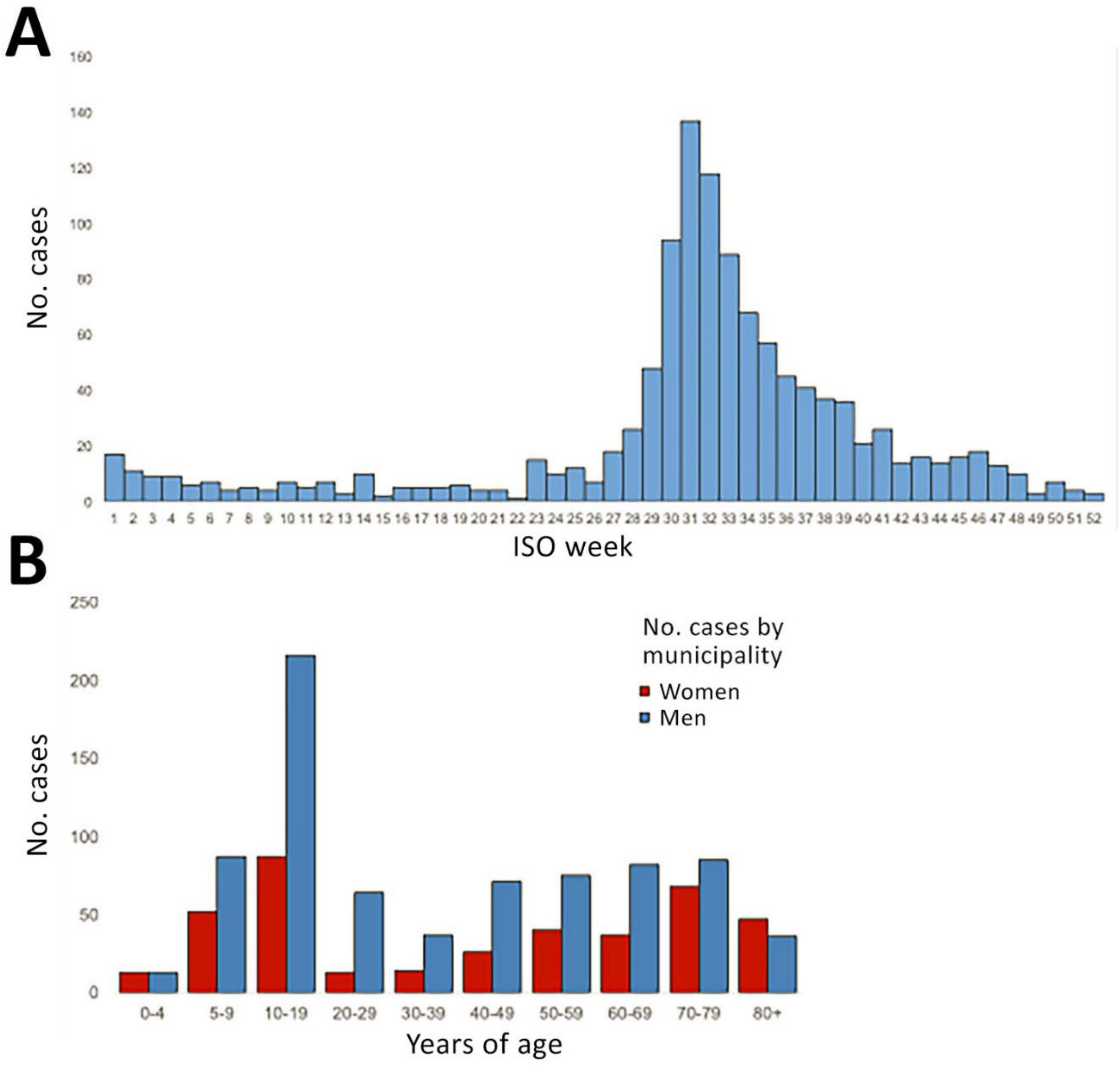

Appendix 1 Figure. Number of patients diagnosed with *Vibrio* spp. and *Shewanella* spp. per ISO week number (panel A) and by sex and age group (panel B) in Denmark during 2010–2018.
